# Beyond Race and Place: Distal Sociological Determinants of HIV Disparities

**DOI:** 10.1371/journal.pone.0091711

**Published:** 2014-04-17

**Authors:** Max-Louis G. Buot, Jeffrey P. Docena, Brenda K. Ratemo, Matthew J. Bittner, Jacob T. Burlew, Aziz R. Nuritdinov, Jennifer R. Robbins

**Affiliations:** 1 Department of Mathematics and Computer Science, Xavier University, Cincinnati, Ohio, United States of America; 2 Department of Biology, Xavier University, Cincinnati, Ohio, United States of America; Alberta Provincial Laboratory for Public Health/University of Alberta, Canada

## Abstract

Informed behavior change as an HIV prevention tool has yielded unequal successes across populations. Despite decades of HIV education, some individuals remain at high risk. The mainstream media often portrays these risk factors as products of race and national borders; however, a rich body of recent literature proposes a host of complex social factors that influence behavior, including, but not limited to: poverty, income inequality, stigmatizing social institutions and health care access. We examined the relationship between numerous social indicators and HIV incidence across eighty large U.S. cities in 1990 and 2000. During this time, major correlating factors included income inequality, poverty, educational attainment, residential segregation and marriage rates. However, these ecological factors were weighted differentially across risk groups (e.g. heterosexual, intravenous drug use, men who have sex with men (MSM)). Heterosexual risk rose significantly with poor economic indicators, while MSM risk depended more heavily on anti-homosexual stigma (as measured by same-sex marriage laws). HIV incidence among black individuals correlated significantly with numerous economic factors but also with segregation and imbalances in the male:female ratio (often an effect of mass incarceration). Our results support an overall model of HIV ecology where poverty, income inequality and social inequality (in the form of institutionalized racism and anti-homosexual stigma) have over time developed into synergistic drivers of disease transmission in the U.S., inhibiting information-based prevention efforts. The relative weights of these distal factors vary over time and by HIV risk group. Our testable model may be more generally applicable within the U.S. and beyond.

## Introduction

“Know your epidemic” was the charge given by the 2007 Joint United Nations Programme on HIV/AIDS, as it noted that the human behaviors promoting HIV transmission are significantly influenced by cultural and structural variations within and across societies [1]. A significant characteristic of HIV incidence in the United States is its racial disparity, with black and Hispanic individuals bearing a disproportionate burden of new infections [2]. Variations in sexual behaviors between black and white individuals (e.g. partner numbers, age of sexual debut) cannot fully explain this [3], [4], [5], [6].

Poverty has emerged as a major force in promoting the transmission of HIV around the world [7], [8], [9]. In the U.S., poverty and HIV are associated [10], [11], [12], and impoverished urban areas have HIV prevalence rates equivalent to those of many low-income countries with generalized epidemics [11]. However, the strength of the connection between poverty and HIV has recently been called into question, as HIV prevalence rates have been found to positively correlate with wealth within some sub-Saharan African countries [13], [14], [15]. Income inequality, however, has remained a stable predictor of HIV across nations, though why remains poorly understood [7], [16], [17].

Socioeconomic status can explain a significant degree—but not all—of the U.S. racial disparities in sexually transmitted infections such as HIV [10], [18]. Hogben and Leichliter [19] have proposed residential segregation as an underlying social determinant of multiple other disparities that increase HIV incidence, including reduced health care access, higher incarceration rates and stigma. Economic instability and male:female ratios skewed by male incarceration may contribute to risky concurrent partnerships [20], [21], [22], [23].

A growing body of literature supports the need to understand how HIV epidemics change over space and time [24]. The U.S. epidemic began in the subpopulation of men who have sex with men (MSM), a group that still accounts for a slim majority of HIV infections [2], [25], yet has proportionately declined over the past two decades. Young, non-white and poor MSM remain particularly at risk [26]. Some have proposed that covert (“down low”) MSM activity is partially responsible for this discrepancy, though this remains a subject of controversy [27], [28].

Much of what we know about the ecology of HIV risk comes from time- and personnel-intensive individual interviews and testing in selected small but representative populations. These are important for causally linking distal and proximal sources of risk. However, gross surveys of whole populations in broad geographical areas have also yielded useful information [12], [29]. We analyzed HIV incidence as a function of differences in “place”—socioeconomics, residential segregation, family structure, health care access, crime rates, male:female ratios and attitudes toward MSM behavior—across eighty U.S. cities, using publicly available data. Here we develop an overall model of HIV ecology that connects easily measurable, distal population-level factors with difficult-to-study proximal risky behaviors, considering both the population at large and marginalized subpopulations. We further subdivide risk behaviors by transmission mode and determine the relative weights of distal socioeconomic factors in promoting HIV incidence amongst heterosexual men, heterosexual women, MSM and intravenous drug users (IDU).

## Methods

### Ethics statement and human subjects

This study was conducted according to the principles expressed in the Declaration of Helsinki. De-identified human subject data was retrieved from the publicly available CDC Wonder database (http://wonder.cdc.gov), which is based on surveillance reports. To maintain consistency with and between sources (mainly U.S. Census and CDC), we have used the term “black” to mean “black or African American,” and “black, not Hispanic.” We have used “white” to mean “white, not Hispanic.” We have used the term “men who have sex with men” (MSM) instead of “homosexual” or “gay” when referring to men who engage in homosexual behavior but may or may not self-identify as homosexual or gay. As the paper argues, we do not believe any of these categories are sufficient to define HIV risk; socioeconomic status plays a greater role.

### HIV Incidence

Cities with populations >100,000 in both 1990 and 2000 were selected for analysis if they were reported as discrete places (cities) by the Centers for Disease Control and Prevention (CDC, Atlanta, GA) and the U.S. Census Bureau (Washington, D.C.). HIV incidence was calculated by first averaging the annual number of cases (all case definitions) from the CDC Wonder database [30] in a five year window centered on the indicated decennial census year to smooth annual fluctuations in small numbers. The average for each city was then divided by that city's total population for that year [31]. Two outliers with incidence >3 standard deviations (SDs) above the mean HIV incidence were removed from analysis (Columbia, SC for 1990, and Fort Lauderdale and Miami, FL for both 1990 and 2000).

For HIV exposure categories, HIV incidence was calculated in the above manner, but using only the CDC-reported cases [30] for the given single exposure category (heterosexual contact, MSM contact, or intravenous drug use (IDU)). When gender was included, the HIV incidence denominator remained the total city population.

For “calibrated” incidence rates concerning race, the numerator was the total number of HIV reports on black or white individuals, and the denominator was the total number of black or white individuals for that city, respectively, in 2000 [31]. For “calibrated” incidence rates concerning MSM exposure, the numerator was the number of MSM-exposed individuals (single exposure category) and the denominator was the estimated number of gay, lesbian or bisexual (GLB) individuals for that city, as calculated by multiplying the estimated percentage of GLB individuals in a city's congressional district(s) [32] by the city's total population. Four outliers with values >3 SD were removed from analysis of MSM calibrated incidence (Columbia, SC; Detroit, MI; Fort Wayne, IN; New Haven, CT).

### Other metrics

All data was collected at the Census city (not metropolitan statistical area) level unless otherwise noted. Household Gini coefficients were calculated using the method described by Glaeser et al. [33]. Segregation indices were taken from the American Community Project using 1990 and 2000 Census data [34]. The living wage estimates were provided by the MIT Living Wage Calculator [35]. Health insurance estimates were obtained from The Commonwealth Fund [36]. Crime indices were reported by the Federal Bureau of Investigation [37]. The anti-MSM stigma scale was created by analysis of laws pertaining to same-sex marriage (SSM) as of July, 2013, with states where SSM was legal receiving a score of 1 and states where SSM was uniformly banned receiving a score of 3. States with civil unions (with or without coincident constitutional SSM bans) or no SSM legislation were given a score of 2.

### Data analysis

The average HIV incidence for each independent variable's highest and lowest quartiles was calculated, and the ratio between them—the association factor—determined. Student's T-tests were used to assess statistical similarity between the HIV incidences in highest and lowest quartiles. Because the sample size in these populations was relatively small (n = 20 per quartile), we confirmed their validity using a bootstrap analysis in which we randomly sorted all cities' HIV incidences and calculated the ratio of the average incidences in resulting top and bottom quartiles. This was performed 100 times in order to generate a distribution of ratios. The resulting *p* values were very similar to those generated by the more traditional T tests (data not shown).

Principle component analysis was performed using DeltaPlot software for Mac [38]. Other data analysis used R [39] or Microsoft Excel (Redmond, WA) software.

## Results

**Figure 1:**
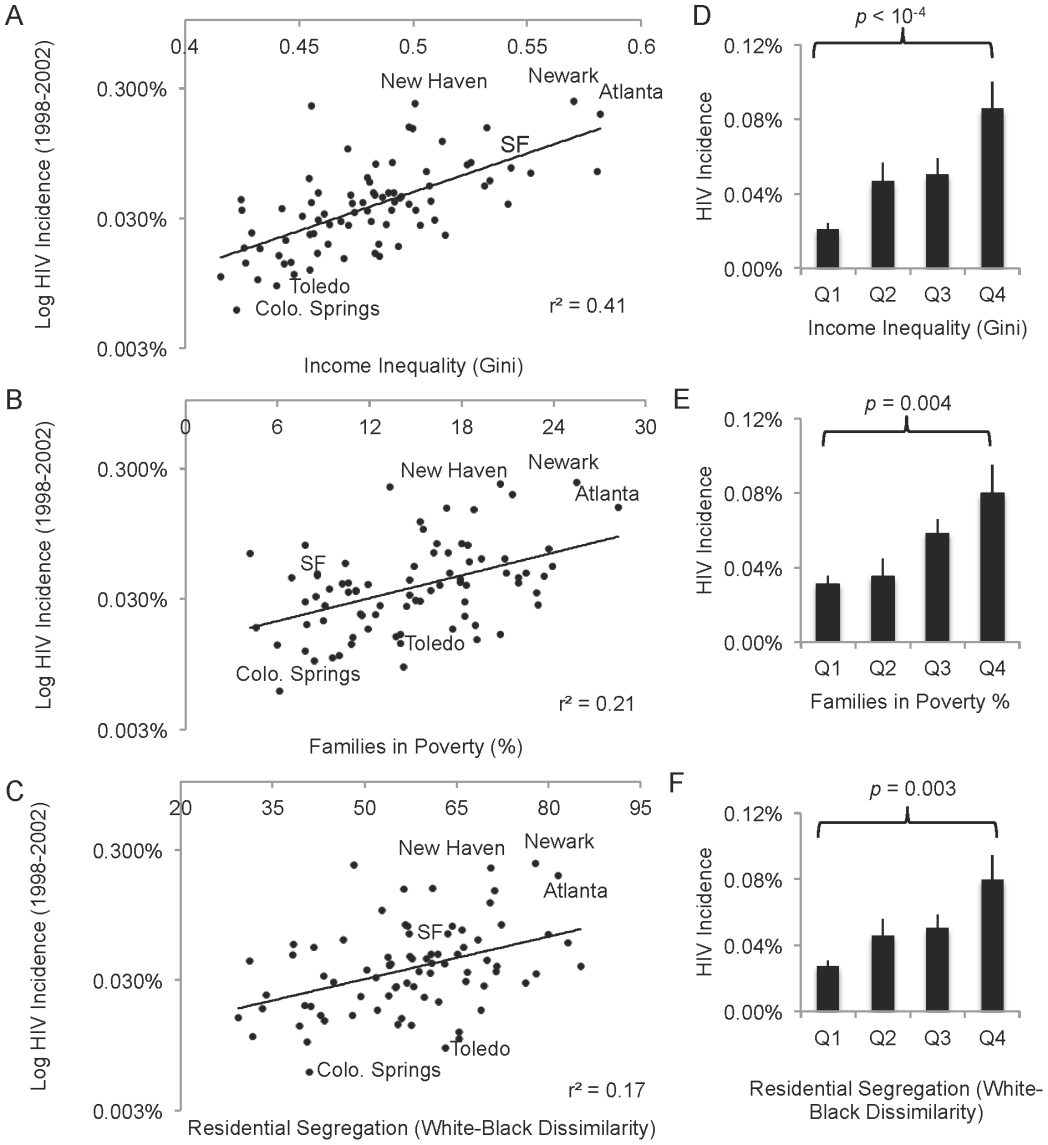
Income inequality, segregation and poverty positively and synergistically correlate with 1998-2002 HIV incidence across 80 U.S. cities. **A–C.** The total reported HIV incidence in 80 large U.S. cities as a function of: (A) income inequality (represented as the Gini coefficient, where higher values correspond to greater inequality); (B) poverty, and (C) black-white dissimilarity index, a measure of black-white segregation where 0 is completely integrated and 100 completely segregated; and. Cities high in all three social determinants tend to be significantly higher in HIV incidence and vice versa (SF = San Francisco). **D–F.** Cities were sorted by (D) income inequality, (E) poverty or (F) black-white segregation, and the HIV incidence averaged over quartiles, where Q1 represents the average HIV incidence for the 20 cities with the highest income inequality and Q4 the average HIV incidence in the 20 cities with the lowest income inequality. T-test comparison of the HIV values in first and fourth quarters illustrates these populations are statistically distinct (see Table 1). Bars = SEM.

We compared average HIV incidence across 80 U.S. cities, and found that income inequality was a significant predictor of HIV incidence in 2000 (r^2^ = 0.41; p<10^−5^ for T-test of first vs last quartiles; Figure 1A,D), as has been previously demonstrated at the global level [7]. Poverty was a weaker but still significant associating factor (r^2^ = 0.21; p = 0.004 for T-test of first vs last quartiles; Figure 1B,E). A third major factor, more particular to the U.S., was racial segregation of black individuals (r^2^ = 0.17; p = 0.003 for T-test of first vs last quartiles; Figure 1C,F). Cities that were high in all three of these categories tended to have above-regression HIV incidence in all three; the reverse was also true.

**Figure 2:**
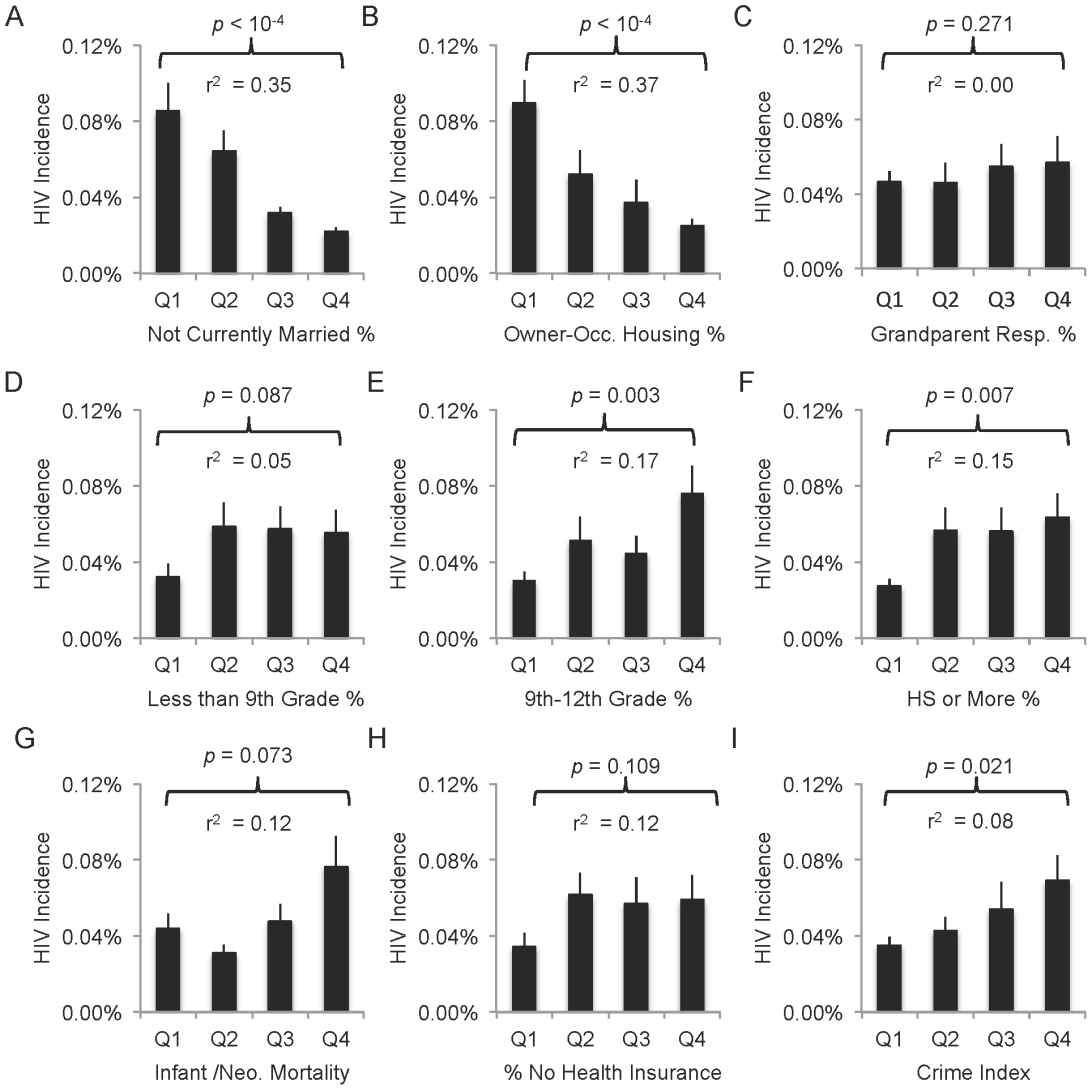
Examples of possible degrees of population-level HIV/socioeconomic associations. Cities were sorted by various 2000 population metrics (x-axes), and the HIV incidence averaged over quartiles, where Q1 represents the average HIV incidence for the 20 cities with the highest values for that census metric and Q4 the average HIV incidence in the 20 cities with the lowest values for that census metric. T-test comparison of the HIV values in first and fourth quarters illustrates that many metrics are associated with HIV incidence (A, B, E, F, I), while others weakly associate (D, G, H) and still others are not likely to be associated (C). Bars = SEM.

HIV incidence associated to various degrees with many other socioeconomic and demographic indicators in U.S. cities (examples in Figure 2). Correlation coefficients (r^2^) between log HIV incidence and each characteristic were estimated, but for some metrics (e.g., education), the strength of the relationship eroded above some threshold minimal value. We therefore determined that comparison of high and low cities for each characteristic was merited. An HIV association factor representing the ratio between average HIV incidence rates in the highest and lowest quartiles for each metric was calculated; effectively, this represents an odds ratio between the populations in the highest and lowest groups of cities for that metric (Figure 2; Table 1). Differences in HIV incidence rates were statistically determined from a ratio T-test for unequal variances in the two quartile groups [40].

**Table 1:** Urban HIV incidence as a function of socioeconomic and demographic metrics of 80 U.S. cities in 2000 (unless otherwise indicated).

	Average	SD	Q1 Ave	Q4 Ave	Q1: HIV×10^−5^	Q4: HIV×10^−5^	HIV Assoc. Factor*	90% CI	p (Q1:Q4 T-test)
**Economic**									
**Income & Expenses**									
Income Inequality (Gini)	0.481	0.036	0.438	0.529	21	86	**4.07**	2.72–5.94	**0.000**
Median Household Income*	$35,969	$7,859	$27,704	$45,564	73	33	**2.22**	1.41–3.28	**0.015**
Living Wage (2012)	$19,875	$2,693	$17,211	$23,715	24	98	**4.00**	2.76–5.63	**0.000**
**Poverty**									
Families Living in Poverty	14.8%	5.40%	8.04%	21.74%	31	80	**2.57**	1.63–3.85	**0.007**
Female Householders in Poverty	31.0%	7.43%	21.12%	39.77%	31	73	**2.32**	1.50–3.42	**0.009**
**Opportunity/Hope**									
Owner Occupied Housing*	50.2%	9.52%	37.53%	60.61%	86	25	**3.45**	2.18–5.36	**0.001**
Vacant Housing	7.95%	2.91%	4.57%	11.95%	43	68	1.59	1.00–2.40	0.103
Unemployment	4.92%	1.49%	3.43%	7.01%	34	84	**2.45**	1.45–4.56	**0.011**
**Education**									
Less than 9th Grade Education	8.28%	3.90%	4.40%	13.85%	32	55	1.73	1.00–3.01	0.102
9th–12th Grade Education	14.1%	4.15%	8.96%	19.36%	31	76	**2.50**	1.62–3.69	**0.006**
High School or More*	77.7%	6.70%	69.11%	85.66%	64	27	**2.33**	1.50–3.43	**0.010**
College or More	25.9%	9.34%	15.80%	37.78%	57	55	0.97	0.59–1.69	0.928
**Health**									
Infant/Neo. Mort. (×10^−5^) (1999)	29.8	16.2	16.08	48.22	45	80	1.76	1.07–2.79	0.072
% Uninsured (1997)	16.8%	5.98%	11.41%	25.15%	35	50	1.42	0.88–2.22	0.217
**Residential Segregation**									
White-Black Dissimilarity	57.4	12.9	39.88	73.28	37	78	**2.12**	1.21–4.44	**0.033**
Black Isolation	48.6	25.08	13.86	79.12	27	76	**2.84**	1.92–4.04	**0.002**
**Family Structure**									
Currently Married*	42.2%	6.69%	33.88%	51.17	86	22	**3.92**	2.65–5.48	**0.000**
Never Married	36.4%	6.29%	28.60%	44.76	22	92	**4.25**	2.85–4.99	**0.000**
Grandpar. Resp. for Grandchild.	45.6%	7.68%	35.14%	54.28%	46	57	1.22	0.67–1.89	0.519
**Crime Index** (per 100,000)	7,317	2,065	4,898	9,981	36	70	**1.94**	1.23–2.82	**0.031**
**Ethnicity**									
%White*	55.0%	16.3%	32.84%	73.36%	87	23	**3.78**	2.54–5.47	**0.000**
%Black	27.8%	19.7%	6.62%	55.04%	29	79	**2.70**	1.83–3.87	**0.001**
%Hispanic/Latino (any race)	15.7%	15.3%	2.26%	37.34%	53	48	0.91	0.49–1.48	0.740
**City Size**									
Population	641,973	1,021,194	162,982	1,621,498	66	44	0.66	0.43–1.12	0.182

Low (Q1) and high (Q4) quartiles for each metric are shown, with their 1998–2002 HIV incidence averages. HIV association factor is the ratio between HIV incidences for Q4 and Q1, or, for starred metrics (*), Q1:Q4* (for easier comparison where a smaller metric value theoretically predicts greater risk). Boldfaced = p<0.05).

High income inequality, low incomes, high unemployment, high poverty, low home ownership and high cost of living (living wage) all correlated positively with HIV incidence, increasing risk by 2–3-fold (Table 1). Additionally, cities with fewer high school graduates and higher segregation experienced similar rises in HIV (Table 1). Indicators of black segregation correlated (Table 1); Hispanic segregation did not (data not shown). Rates of marriage were even more highly correlated, with low-marriage-rate cities experiencing ∼4-fold higher HIV rates.

In 1990, the association between social conditions and HIV in U.S. cities was less profound (Table 2). However, income inequality, poverty and black segregation remained significant associating factors.

**Table 2:** Urban HIV incidence as a function of socioeconomic and demographic metrics of 80 U.S. cities in 1990.

	Average	SD	Q1 Ave	Q4 Ave	Q1: HIV×10^−5^	Q4: HIV×10^−5^	HIV Assoc. Factor*	90% CI	p (Q1:Q4 T-test)
**Economic**									
**Income & Expenses**									
Income Inequality (Gini)†	0.469	0.049	0.410	0.532	25	82	**3.33**	1.95–5.07	**0.008**
Median Household Income*	$25,921	$4,843	$20,427	$32,170	63	68	0.93	0.45–1.70	0.829
**Poverty**									
Families Living in Poverty	15.9%	5.82%	9.18%	23.32%	27	70	**2.58**	1.39–4.15	**0.031**
Female Householders in Poverty	8.99%	5.16%	2.96%	15.94%	59	74	1.26	0.66–2.28	0.509
**Opportunity/Hope**									
Owner Occupied Housing*	44.6%	8.29%	33.28%	53.72%	121	22	**5.62**	3.85–8.01	**0.000**
Vacant Housing	9.21%	2.84%	5.81%	12.95%	40	64	1.62	0.81–2.74	0.215
Unemployment	5.10%	1.50%	3.53%	6.90%	51	79	1.55	0.80–3.08	0.255
**Education**									
Less than 9th Grade Education	10.6%	3.99%	6.06%	15.79%	40	65	1.63	0.86–2.83	0.187
9th–12th Grade Education	16.2%	4.56%	10.85%	22.02%	42	74	1.76	0.90–3.79	0.157
High School or More*	73.5%	7.46%	64.24%	82.68%	70	36	1.91	1.12–2.91	0.062
College or More	22.2%	7.99%	13.81%	31.79%	49	77	1.56	0.85–3.50	0.225
**Health**									
Infant/Neo. Mort. (×10^−5^) (1999)	29.6	16.3	16.08	47.94	67	71	1.07	0.56–1.93	0.841
% Uninsured (1997)	16.7%	6.01%	11.41%	25.15%	35	65	**1.84**	1.10–2.89	0.061
**Social**									
**Residential Segregation**									
White-Black Dissimilarity	62.1	12.98	44.99	77.41	35	83	**2.39**	1.28–4.97	**0.032**
Black Isolation	52.9	24.53	17.70	80.78	31	80	**2.55**	1.47–3.92	**0.020**
**Crime Index** (per 100,000)	9,463	2,591	6,568	12,927	31	91	**2.91**	1.66–4.65	**0.013**
**Ethnicity**									
%White*	61.6%	17.3%	37.27%	80.19%	90	25	**3.57**	2.16–5.73	**0.003**
%Black	27.2%	19.3%	7.31%	54.27%	41	78	1.94	1.00–4.31	0.098
%Hispanic/Latino (any race)	11.1%	13.1%	0.95%	29.58%	42	63	1.48	0.74–2.85	0.309
**City Size**									
Population	583,946	928,459	166,655	1,424,425	52	65	1.26	0.72–2.24	0.478

Low (Q1) and high (Q4) quartiles for each metric are shown, with their 1988–1992 HIV incidence averages. HIV association factor is the ratio between HIV incidences for Q4 and Q1, or, for starred metrics (*), Q1:Q4* (for easier comparison where a smaller metric value theoretically predicts greater risk). When the p value is significant (p<0.05), the association factor is boldfaced. Each metric is from the year 1990 unless otherwise indicated.

† Internally comparable, but not comparable to 2000 Gini; income brackets in 1990 Census were different.

### Exposure modes

**Figure 3:**
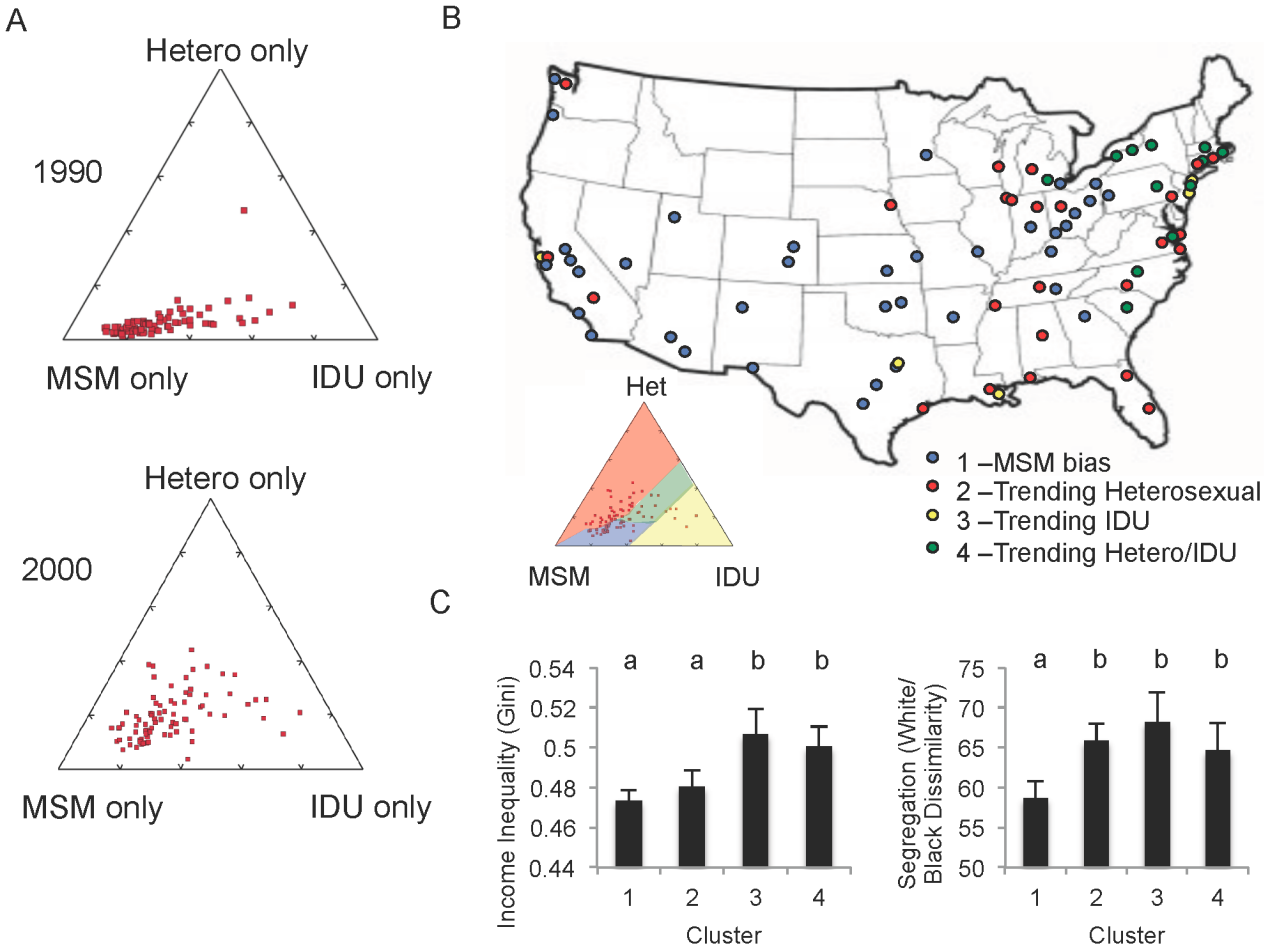
Proportion of HIV reports in each city with exposure risk of male-male sexual contact (MSM), heterosexual contact (Het.) or IV drug use (IDU). (Multiple or unknown exposure categories excluded.) **A.** Principle component analysis for HIV cases 1988–1992 (top) and 1998–2002 (bottom) shows an overall national trend away from MSM and toward heterosexual and/or IDU exposures. **B.** Cities clustered based on exposure trends. MSM-biased cluster (blue) is approximately delimited by the 1990 MSM region of the PCA plot. **C.** Average socioeconomic metrics were sorted with exposure clusters. More segregated cities (measured by white/black dissimilarity index) were significantly more likely to experience a higher proportion of non-MSM HIV cases. Cities with high income inequality were more likely to report a higher proportion of IDU-associated HIV. Lowercase letters represent statistical similarity (by T-test, p<0.05).

The 1990–2000 shift toward greater dependence of HIV incidence on socioeconomics was coincident with a shift away from MSM behaviors as the major mode of acquisition; heterosexual and IDU exposure rose in many cities (Figure 3A). To test whether the two shifts were related, we sorted cities into pattern clusters based on their 2000 deviation from the observed 1990 predominately MSM bias (Figure 3B). When the socioeconomic and demographic metrics of these clusters were compared, we found cities that had trended toward IDU and mixed IDU/heterosexual exposure risk had higher income inequality (p<0.03; Figure 3C). Collectively, all the cities trending away from MSM averaged significantly higher segregation (p<0.02; Figure 3C). Other metrics showed similar patterns, but were not statistically significant (p>0.05).

However, when we calculated HIV incidences per city for specific exposure modes, clear differences emerged (Table 3). Rates of infection by heterosexual contact (male and female) were significantly associated with income inequality and poverty indicators as well as education and segregation (p<0.05). HIV association factors were generally similar between males and females, although income inequality increased risk more for males while poverty, health and housing circumstances increased risk more for females. Incidence rates by IDU as the sole exposure were less significantly associated with these indicators, and MSM was not significantly associated with any of the indicators except segregation and marriage (p<0.05; Table 3, column 4).

**Table 3:** Urban HIV incidence by exposure category from 1998–2002 as a function of socioeconomic and demographic metrics of 80 U.S. cities in 2000 (unless otherwise indicated).

	HIV Association Factor (90% C.I.)							
	Female Hetero		Male Hetero		IDU		MSM (Uncalibrated)	
**Economic**								
**Income & Expenses**								
Gini Coefficient (Households)	**4.32**	**(2.52–7.47)**	**5.80**	**(3.34–0.02)**	3.39	(1.24–15.07)	1.89	(1.08–5.29)
Median Household Income*	**3.06**	**(1.67–5.33)**	**2.42**	**(1.24–4.44)**	3.41	(1.19–7.01)	1.08	(0.80–1.53)
Living Wage (2012)	**5.83**	**(3.60–9.20)**	**6.21**	**(3.82–9.57)**	**4.36**	**(1.83–22.50)**	1.82	(1.08–3.94)
**Poverty**								
Families Living in Poverty	**3.64**	**(2.08–6.11)**	**3.42**	**(1.87–6.04)**	2.77	(1.09–7.87)	1.00	(0.59–1.93)
Female Householders in Poverty	**3.77**	**(2.18–6.24)**	**3.69**	**(2.06–6.50)**	3.10	(0.90–6.65)	1.23	(0.79–1.85)
**Opportunity/Hope**								
Owner Occupied Housing*	**3.61**	**(1.99–7.00)**	**2.74**	**(1.42–5.85)**	**4.14**	**(1.75–19.44)**	1.69	(0.94–3.99)
Vacant Housing	**2.02**	**(1.13–3.50)**	1.84	(0.98–3.20)	1.29	(0.59–2.75)	1.15	(0.78–1.65)
Unemployment	**3.18**	**(1.60–9.79)**	**4.99**	**(2.80–8.86)**	**4.45**	**(1.85–9.36)**	1.35	(0.85–2.33)
**Education**								
9th–12th Grade Education	**4.26**	**(2.49–7.02)**	**3.92**	**(2.19–6.89)**	**4.34**	**(1.79–8.84)**	1.25	(0.91–1.81)
High School or More*	**3.45**	**(1.90–5.85)**	**3.19**	**(1.67–5.79)**	2.88	(0.97–10.27)	0.94	(0.60–1.90)
**Health**								
Infant/Neo. Mort. (×10^−5^) (1999)	**2.33**	**(1.25–4.38)**	1.91	(0.94–3.71)	2.05	(0.62–4.22)	1.31	(0.86–1.93)
% Uninsured (1997)	1.23	(0.55–2.31)	0.88	(0.55–1.56)	1.29	(0.58–2.96)	1.18	(0.69–2.07)
**Social**								
White-Black Dissimilarity	**2.35**	**(1.16–7.13)**	1.88	(0.88–6.22)	2.16	(0.66–9.68)	**2.10**	**(1.33–3.27)**
Black Isolation	**4.21**	**(2.55–7.06)**	**3.89**	**(2.19–7.41)**	3.69	(0.95–7.08)	**1.79**	**(1.25–2.54)**
**Family Structure**								
Currently Married*	**5.30**	**(3.13–9.00)**	**4.42**	**(2.55–7.67)**	**7.23**	**(3.13–12.20)**	**2.32**	**(1.65–3.19)**
Never Married	**5.60**	**(3.25–9.54)**	**4.89**	**(2.75–8.44)**	**4.02**	**(1.52–20.61)**	1.74	(1.00–4.28)
Grandpar. Resp. for Grandchild.	1.48	(0.67–2.60)	1.15	(0.51–2.10)	1.09	(0.44–2.20)	1.27	(0.69–1.97)
**Crime Index** (per 100,000)	**2.50**	**(1.40–4.07)**	**2.33**	**(1.27–3.83)**	1.16	(0.57–2.86)	1.58	(1.00–2.33)
**City Size**								
Population	0.59	(0.33–1.10)	0.71	(0.37–1.44)	0.54	(0.28–1.14)	0.68	(0.44–1.19)

HIV association factor is the ratio between HIV incidences for Q4 and Q1, or, for starred metrics (*), Q1:Q4* (for easier comparison where a smaller metric value theoretically predicts greater risk). For each population, HIV incidence was obtained by dividing the total number of HIV cases for that single exposure category by the total population. Boldfaced = p<0.05 for similarity between Q1 and Q4 (Student's T-test).

### Male:female ratios

**Figure 4:**
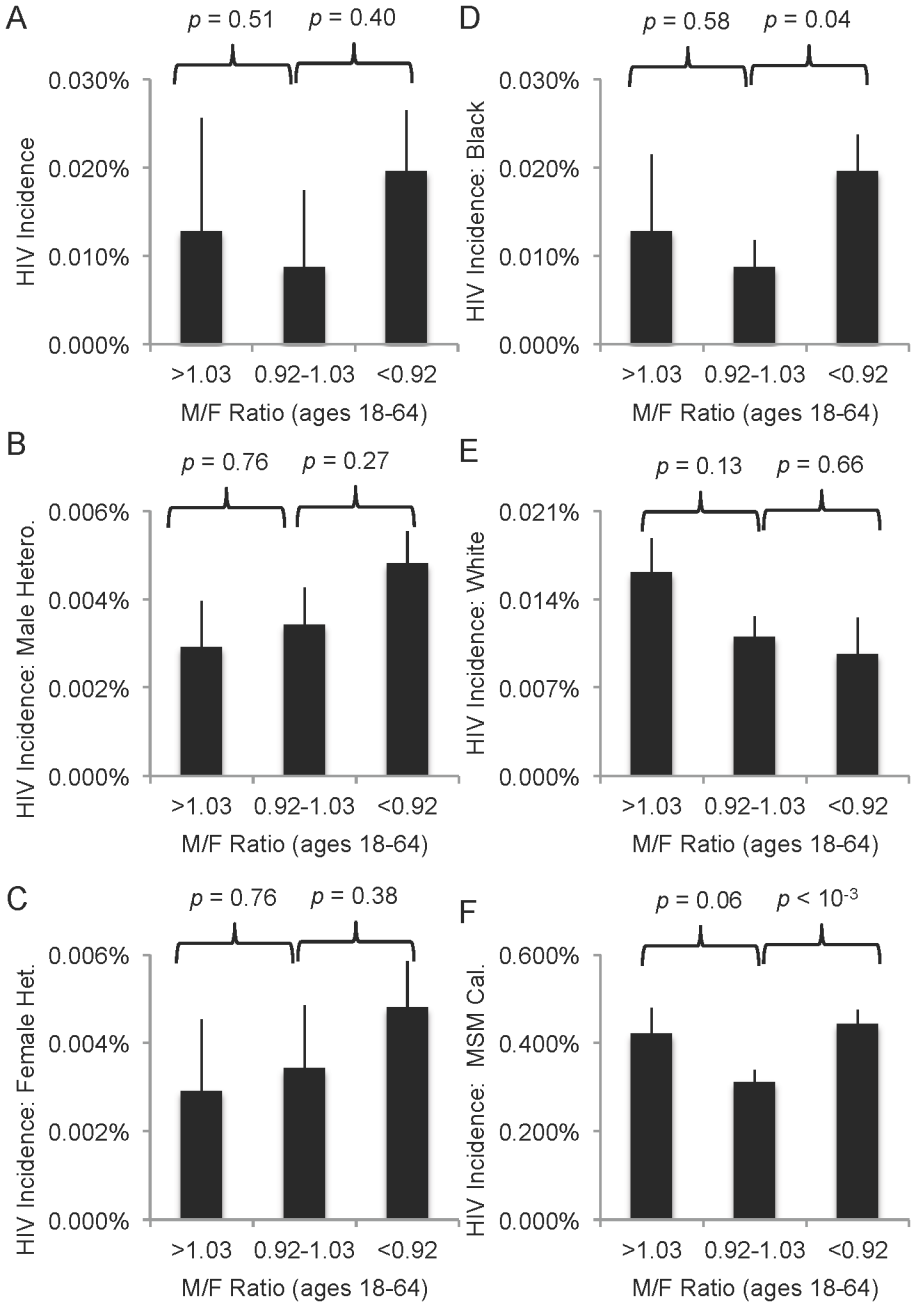
Association of HIV and skewed male:female ratios supports effect of such ratios on partner concurrency. M:F ratios were calculated as the total number of male individuals aged 18–64 divided by the total number of female individuals aged 18–64 for each city. Groups were established as deviating >0.05 from the mean M:F ratio (0.97) and *p* values are for T-tests between extremes (n = 24 for <0.92 and 13 for >1.03) and mid-range (n = 43). **A.** Total reported HIV incidence per city varies by M:F ratio, but not significantly. **B–C.** HIV incidence reports amongst (**B**) males and (**C**) females exposed by heterosexual contact. **D–E.** HIV incidence among (**D**) black or (**E**) white individuals. **F.** HIV incidence reports by MSM exposure only, as a percentage of estimated number of GLB individuals per city. Bars = SEM.

Another important demographic characteristic is the male:female ratio, which sometimes falls well below 1 in cities with high incarceration rates or rises above 1 where major economic industries disproportionately attract men (e.g. cities near military bases). Other studies have documented how skewed ratios increase partner concurrency, a major driver in sexual HIV transmission [20], [22], [23]. We calculated each city's 2000 male:female ratio for adults aged 18–64 and separated male- and female-biased clusters defined as deviating ±0.05 from the mean (0.97; Figure 4). Though not statistically significant, the imbalanced clusters had higher HIV incidences (Figure 4A), and in cities with many more females than males, heterosexual risk was higher for both genders (Figure 4B–C).

Black men are disproportionately incarcerated or victims of violent crime [1], leaving behind communities with more women than men [22], [23]. Thus, one might expect that black individuals are disproportionately affected by skewed male:female ratios. We calculated HIV incidence rates for black individuals in each city (calibrated rate). Indeed, HIV incidence was significantly higher for black individuals in cities with low M:F ratios (Figure 4D). A different pattern was observed for white individuals, who experienced higher (but not statistically significantly higher, *p* = 0.13) risk where M:F ratios were high (Figure 4E).

### Disproportionate risk and race

Numerous other differences were observed in the risk ecology between black and white individuals. In 2000, HIV incidence was significantly more associated with poor economic indicators for black individuals than for white individuals (Table 4). HIV association factors for all economic metrics ranged from ∼4–15 fold amongst black individuals (Table 4), compared to ∼2–4 fold for all (Table 1), with income inequality having the greatest effect. Segregation was associated with a very high increase in risk, consistent with the predictions of Hogben and Lichliter (2008). For white individuals, only home ownership rates were significantly associated with HIV incidence, and then only at ∼1.8-fold (Table 4).

**Table 4:** Calibrated HIV incidence for select populations from 1998–2002 as a function of socioeconomic and demographic metrics of 80 U.S. cities in 2000 (unless otherwise indicated).

	HIV Association Factor (90% C.I.)					
	Black Individuals (Calibrated)		White Individuals (Calibrated)		MSM (Calibrated)	
**Economic**						
**Income & Expenses**						
Gini Coefficient (Households)	**15.00**	**(7.07–31.82)**	1.07	(0.63–2.12)	**2.23**	**(1.81–2.77)**
Median Household Income*	**6.71**	**(3.08–18.18)**	1.49	(0.80–2.37)	**1.49**	**(1.20–1.86)**
Living Wage (2012)	**7.67**	**(3.66–19.28)**	1.56	(0.89–2.71)	**1.42**	**(1.10–1.87)**
**Poverty**						
Families Living in Poverty	**8.49**	**(3.73–23.81)**	1.12	(0.50–2.00)	**1.42**	**(1.12–1.83)**
Female Householders in Poverty	**9.92**	**(4.16–42.40)**	1.09	(0.57–1.86)	**1.49**	**(1.17–1.90)**
**Opportunity/Hope**						
Owner Occupied Housing*	**4.95**	**(1.74–17.20)**	**1.81**	**(1.14–2.71)**	1.35	(1.03–1.80)
Vacant Housing	**5.74**	**(2.60–14.09)**	0.96	(0.58–1.52)	**1.47**	**(1.22–1.79)**
Unemployment	**9.64**	**(4.24–43.33)**	0.86	(0.47–1.46)	**1.76**	**(1.42–2.17)**
**Education**						
9th–12th Grade Education	**8.42**	**(3.87–27.11)**	0.90	(0.51–1.52)	**1.56**	**(1.21–2.04)**
High School or More*	**6.16**	**(2.10–19.86)**	0.78	(0.52–1.30)	**1.51**	**(1.17–2.00)**
**Health**						
Infant/Neo. Mort. (×10^−5^) (1999)	2.52	(1.02–32.52)	0.94	(0.48–1.54)	**1.43**	**(1.13–1.81)**
% Uninsured (1997)	1.39	(0.53–3.41)	1.11	(0.66–1.64)	1.27	(1.01–1.62)
**Social**						
White-Black Dissimilarity	**9.26**	**(4.07–55.98)**	0.80	(0.44–1.58)	**1.72**	**(1.38–2.18)**
Black Isolation	**54.12**	**(30.47–95.66)**	0.71	(0.47–1.15)	**1.73**	**(1.40–2.16)**
**Family Structure**						
Currently Married*	**15.24**	**(7.24–37.06)**	1.58	(0.96–2.48)	**1.44**	**(1.11–1.91)**
Never Married	**14.24**	**(5.74–48.69)**	1.64	(1.03–2.50)	1.33	(0.99–1.79)
Grandpar. Resp. for Grandchild.	**3.41**	**(1.50–7.40)**	0.90	(0.48–1.78)	1.26	(1.00–1.57)
**Crime Index** (per 100,000)	**5.80**	**(2.35–15.95)**	1.23	(0.74–1.88)	1.10	(0.85–1.39)
**City Size**						
Population	0.96	(0.30–2.22)	0.44	(0.29–0.75)	0.94	(0.74–1.19)

HIV association factor is the ratio between HIV incidences for Q4 and Q1, or, for starred metrics (*), Q1:Q4* (for easier comparison where a smaller metric value theoretically predicts greater risk). For each population, HIV incidence was obtained by dividing the total number of HIV cases for that exposure category by the total estimated number of individuals in that category (i.e. number of black, white or LGB individuals). Boldfaced = p<0.05 for similarity between Q1 and Q4 (Student's T-test).

In addition to the differential effects of segregation and poverty in these two populations, black and white HIV incidence was coupled to different proportional risk groups. Across the U.S., CDC reports of HIV exposures in black individuals (1998–2002) were roughly equally split among heterosexual, IDU or MSM. In contrast, ∼70% of cases in white individuals were linked to MSM and only ∼11% to heterosexual contact (only these exposure categories included in analysis, all single risk). This may explain the difference in M:F ratio effects (Figure 4).

### MSM risk

While the number of MSM-linked HIV cases as a fraction of the entire city population was not significantly linked to socioeconomic indicators (Table 3, column 4), this method of incidence calculation assumes that men are equally likely to engage in MSM behavior across all U.S. cities. It is generally recognized, however, that some cities are more accepting of MSM behavior than others, and that individuals who identify as gay, lesbian or bisexual (GLB) are more likely to migrate to those cities. This could artificially inflate the MSM risk in such cities, especially considering the higher risk of HIV transmission involved in anal intercourse (relative to vaginal intercourse) [2]. A more rigorous analysis would calibrate the number of HIV cases linked to MSM exposure to the number of individuals engaging in MSM behavior in each city, or at least to GLB individuals as a proxy.

The 2005 American Community Survey asked for the first time about same-sex couples, and consequently contained enough information to extrapolate estimates of GLB individuals across the country [32]. We used these findings to produce a MSM incidence rate for each city calibrated to the estimated number of GLB individuals in that city's congressional district(s) (Table 4, column 3). These calibrated incidence rates demonstrated that proportional MSM risk is also associated with income inequality, poverty and segregation, though at lower values (∼1.5-fold; Table 4) than heterosexual risk (∼2–6-fold; Table 3). Further, this calibrated MSM incidence rate associated with skewed male:female ratios, with both extremes linked to higher risk (p = 0.06 for male-biased communities and p<10^−5^ for female-biased communities; Figure 4F).

**Figure 5:**
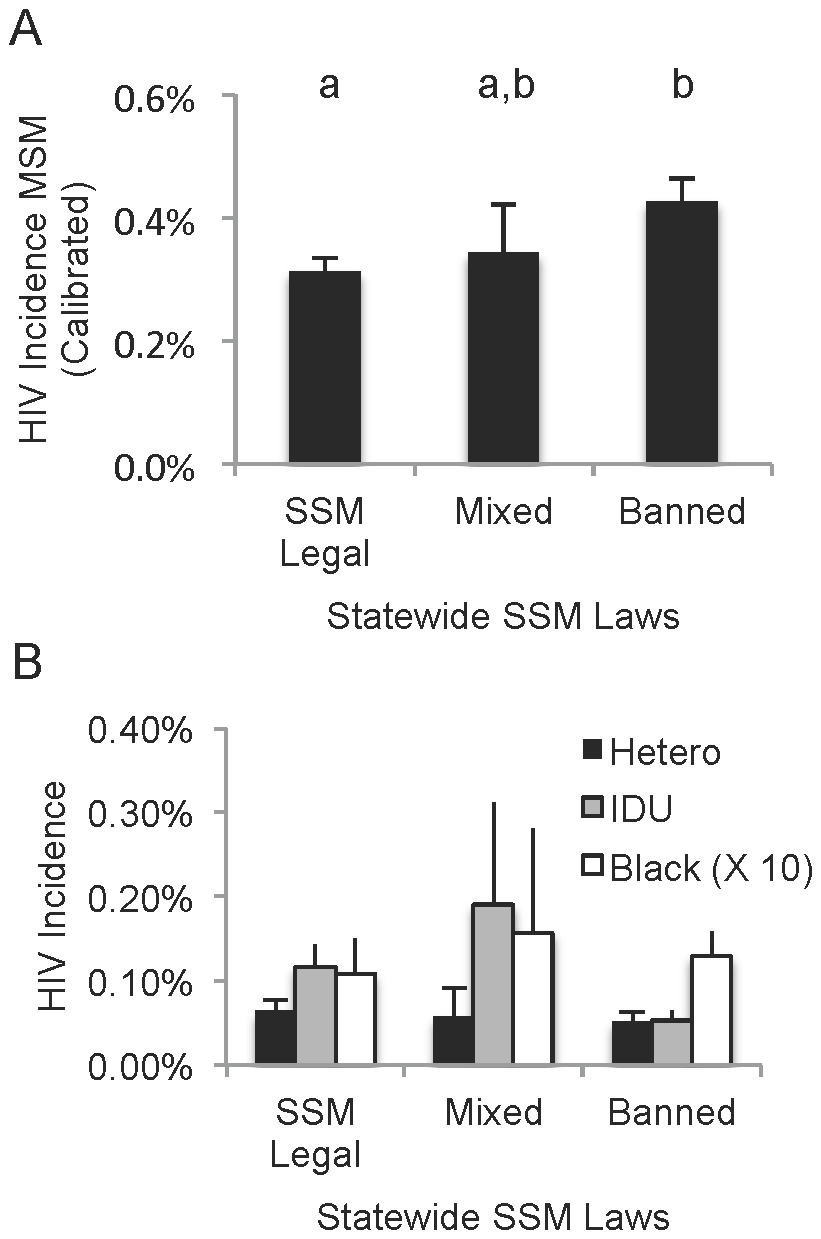
Stigmatization of homosexual behavior (as measured by statewide laws on same sex marriage (SSM)) correlates with increased MSM HIV incidence. Cities were assigned stigma categories based on their state's position on SSM as of July, 2013. States with legal marriage include 22 cities. States with civil unions, enumerated privilege (with or without constitutional SSM bans) or no relevant laws classified as “mixed” (n = 9 cities). States with legislative or constitutional SSM bans (n = 48 cities) were classified as “banned.” **A.** MSM HIV incidence rates represent total HIV reports by single MSM exposure divided by the estimated total GLB individuals per city (from Gates, 2006). Lowercase letters indicate statistically similar populations (by T-test, p>0.05). **B.** No pattern or significant difference was found in any other group (shown: HIV incidence by heterosexual or IDU exposure, and black individuals). Bars = SEM.

Others have speculated that anti-homosexual stigma at the community level may incentivize covert MSM activity, leading to behaviors that increase HIV risk such as more partners and more partner concurrency (both with other males and with females) [41], [42]. In the absence of consistently collected data on attitudes toward homosexuality across U.S. cities, we turned to statewide same-sex marriage (SSM) laws as a proxy for stigma. We assumed that states with fully legal SSM stigmatize MSM less than those with legal bans against SSM. Indeed, we found that cities in low-stigma states experienced significantly less MSM-based HIV incidence than cities in high-stigma states (p = 0.04; Figure 5A).

To rule out the possibility that our stigma measure simply correlates with some other variable that may increase HIV rates in general (e.g. abstinence-only sex education [25], [43], [44]), we checked it against all other socioeconomically-associated HIV exposure categories. No significant pattern was evident (Figure 5B).

We did not expect data collected at the state level to adequately describe conditions within that state's large cities. For several other publically available measures only available at the state level (incarceration rates, infant mortality, various per capita expenses including utilities and health care), metrics were *not* predictive of HIV incidence for the cities within those states (data not shown), presumably because the state data does not adequately describe the city. Therefore, the strength of the stigma-MSM risk correlation (Figure 5A) was striking.

## Discussion

**Figure 6:**
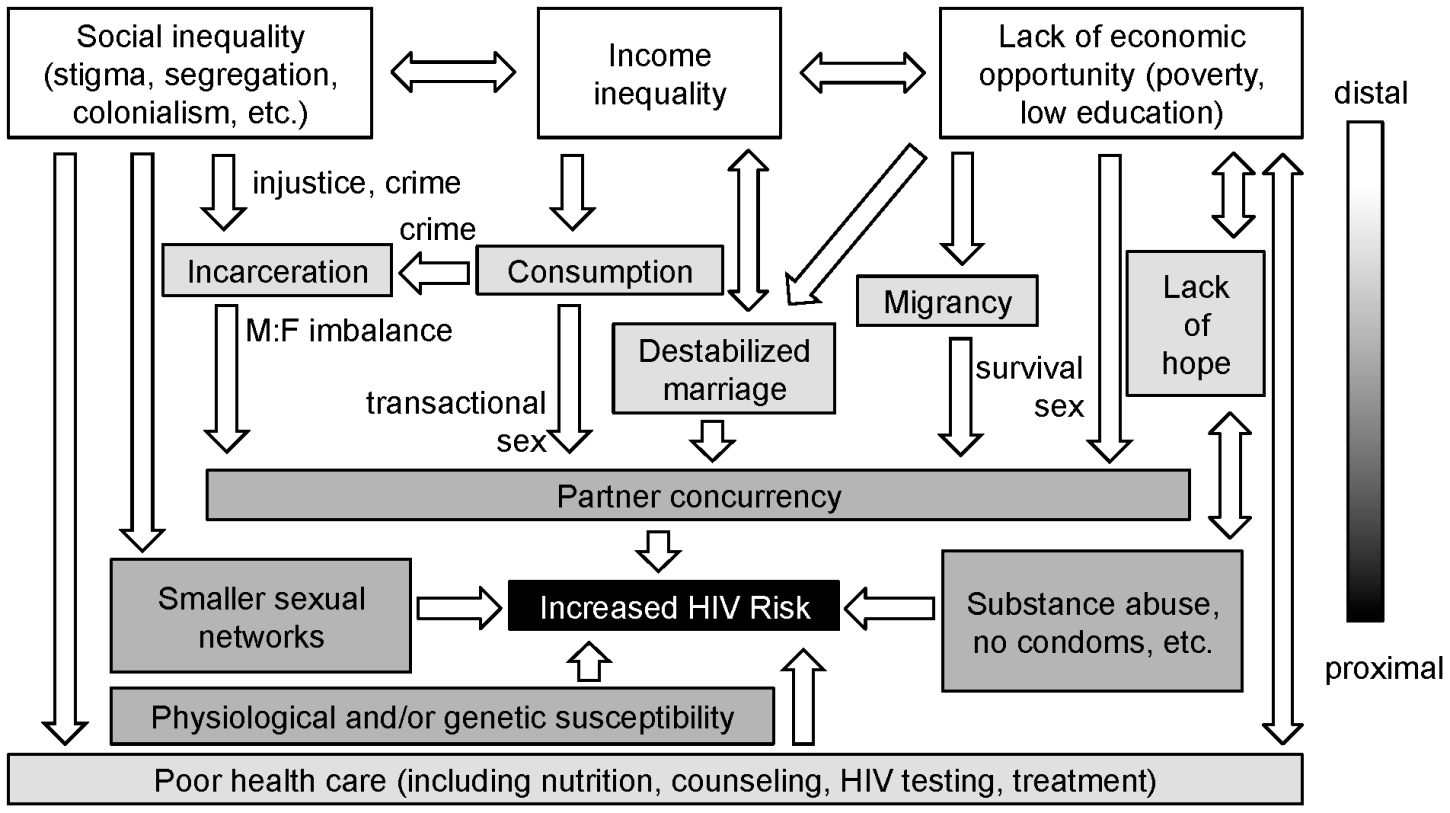
A general model of late-stage epidemic risk. The strongest population-level associations between HIV and social environment, in our studies and other studies, can be reduced to three distinct but interrelated social determinants: social inequality, income income inequality and poverty. These generate a loss of social cohesion as sexual relationships destabilize due to rising material expectations, an inability to meet material needs, gender imbalances and increased expectations for long-term commitment. All of these increase the frequency of concurrent partners. Social inequality restricts sexual networks, increasing the effect of HIV-positive individuals in those networks. Both poverty and inequality decrease access to health care, compromising prevention. Diminished expectations for the future, a common effect of poverty, makes individuals more resistant to the message of behavior change. Finally, the route of transmission matters, with shared needles, anal sex and receptive partners carrying more physiological risk. Differences in genetic susceptibility, little studied, also likely contribute to risk. Individuals experience more risk as the number of biological and sociological determinants they experience increases. Arrows here may be more interconnected than this diagram assumes; however it provides a testable model.

UNAIDs' directive to “Know your epidemic” reminds us that sociocultural context can influence the propagation of HIV through a population [1]. But how granular can or should such analysis be, and how much variation can be expected? Certain ecological factors, found across and within nations, can be variably associated with increased HIV risk; it is possible that mere gross analysis of distal social determinants (social inequality, income inequality and lack of economic opportunity) is adequate to predict risky behaviors and thus HIV incidence. Individual-level sociological studies from around the world can help us connect these distal predictors causally to proximally risky behaviors. We have integrated our findings with numerous other studies to propose a model of underlying universalities in HIV risk that persist beyond region and race (Figure 6).

### Stigma: Men who have sex with men

In the late 1960s [45], HIV emerged in the U.S. amongst MSM, a population in which the virus' preferential propagation was likely due to heterosexual-homosexual differences in behavior (higher numbers of sex partners, lower rates of condom use, smaller sexual networks) and physiological risk (anal intercourse transmits more frequently than vaginal intercourse) [25]. HIV was discovered in 1981 [46], and education and prevention efforts began to show positive effects by the late 1980s [25]. While male homosexual intercourse still accounts for slightly over half of new infections today in the U.S., these are disproportionately among the young and non-white [2], [26].

Why is the behavior change message effective amongst some people and not others? Too often, especially in the public media, the answer stops at race, likely because race is the primary individual-level data that surveillance systems collect. While it is theoretically possible that genetic differences in HIV susceptibility play a role [47] (Figure 6), this is little-studied and unlikely to aid in prevention efforts. The persistent focus on race alone in public health reporting can create and reinforce racism among health care practitioners, their patients and the general public [48].

One's socioeconomic and cultural environment significantly influences one's behavior, regardless of race. Our data show that MSM-associated HIV incidence has shifted over time to communities that stigmatize MSM behavior, and, to our knowledge, it is the first to show such a link at the population level. Presumably, stigmatization encourages covert, short-term sexual encounters with men concurrent with overt relationships with women. This “down low” phenomenon has been dismissed as an explanation for racial disparities in HIV [49], but the literature is conflicted on whether internalized homophobia encourages risk among MSM individuals of any race. Psychosocial studies of HIV clinic workers [41] and small groups of MSM volunteers [42] have found that internalized homophobia does increase risky behavior. However, these small studies have been criticized for sample bias [50], [51], while meta-analysis finds a weak association that is difficult to separate from interrelated risk factors like substance abuse [51]. Our indirect, population-level generalization of these direct, individual-level studies supports the assertion that fighting MSM stigma (not just HIV/AIDS stigma) has an appropriate role in HIV prevention.

### Social Inequality

Stigma may be viewed as a form of social inequality. It can be directed against sexual orientation, but also against race or ancestral origin. Neither individual behaviors nor socioeconomic status alone can explain the higher HIV incidence amongst black individuals in the U.S. [6], [12], [20], [21], leading to the emerging theory that institutional and structural racism is a neglected contributing factor [19]. Our data supported this. We found that residential segregation (white-black dissimilarity and black isolation) was a very strong predictor of HIV incidence amongst black individuals. Others have found the same for gonorrhea [52]. Amongst MSM and for sexually-exposed females, segregation was more highly associated with HIV than were economic factors. Why?

Individual-level studies tell us that residential segregation contributes to smaller sexual networks [21], [53] in which HIV can propagate more quickly. Health status may also contribute: the inequality perceived by both the marginalized and the empowered can result in fewer and more negative contacts with the health care system [19], [48] (Figure 6). We were unable to obtain consistent population-level data on health care access; the indicators we did find showed weak associations for some populations, notably women and MSM. These populations are linked in their higher physiological vulnerability to transmission as potential receptive partners—preventative care may matter more in these groups.

In the U.S., residential segregation is a legacy of slavery and racism, but similar social inequality be seen in other countries with histories of colonial, institutionalized racism (e.g. South Africa) [17]. Any marginalized population may experience the same proximal effects of economic and sexual exclusion. For example, while Ghana has a relatively low national HIV prevalence rate (∼1%), the Krobo ethnic minority experienced HIV prevalence nearly 15-fold higher after forced relocation due to the building of a dam [54]. We therefore termed this distal determinant “social inequality” (Figure 6).

### Economic Opportunities: Poverty and Education

Poverty reduction is often cited as a structural strategy for preventing HIV transmission [8], [16]. However, the universality of the relationship between poverty and HIV has recently been challenged [13], [55] and is clearly more complex than initially assumed [14], [56]. Within sub-Saharan Africa, higher-income individuals may experience more risk (predominately heterosexual) early in an epidemic because increased mobility promotes partner concurrency—later, that wealth may become protective as the prevention message permeates and treatment is accessed [14], [55], [56]. And sub-Saharan African HIV epidemics often begin amongst those with more education but over time shift to those with less education [56].

We found evidence that the same was true across U.S. cities from 1990 to 2000. As in sub-Saharan Africa, HIV incidence was initially highest amongst higher-income, more educated individuals (in this case MSM individuals), but transitioned by 2000 to a poverty- and low-education-dependent risk (Figure 6). This yields a population-level view of the well-documented behavioral changes in the relatively highly educated and wealthy “gay community,” changes that did not take as strong a hold amongst the poor and less educated [25]. We found that the importance of poverty and education level was weak amongst MSM but strong amongst heterosexuals, especially women.

Why might poverty make individuals refractory to behavior modification? In both high- and low-income countries, economic insecurity increases survival sex, in which women or men exchange long-term HIV risk (multiple partners, possibly no condoms) for short-term financial help in meeting their and their family's needs [57], [58], [59]. We see evidence for this in our study, in that home ownership, education and unemployment all correlate with HIV (Figure 6).

Further, lack of economic opportunity also promotes people, particularly men, to migrate in search of better prospects, promoting concurrent partnerships by forming sexual “bridges” between the home and the site of migration [17], [60], [61], [62], [63]. Our analysis of male:female ratios suggests that male labor migration may also increase risk in the U.S., as cities with more men had higher HIV incidence in multiple exposure categories.

As for educational attainment, Hargreaves et al. (2008) speculates that the trend in Africa toward an association between HIV and limited schooling could be due to a longer time spent hearing the prevention message in the classroom [56]. This may be correct, but protective educational attainment levels in African countries are lower (often only concerning primary school) than in the U.S., where we found strong associations with secondary school completion; further, the prevention message is inconsistently disseminated in U.S. schools [43], [44]. It is therefore possible that education is additionally protective—universally so—when it provides increased economic opportunity. Hopelessness for future improvements in quality of life has been proposed as a major modulator of risky behaviors such as substance abuse (including IDU) and unprotected sex [64]. In our data, low home ownership and high unemployment might not only signify economic insecurity, but also hopelessness (Figure 6)—these were strongly correlated with all HIV exposure groups except for MSM, and, along with low educational attainment, were prominent predictors of IDU risk.

### Income Inequality

Income inequality is significantly associated with HIV incidence and prevalence across countries, even more so than poverty [7], [16]. Our study verifies that the same trend can be found within a country at the level of large cities; in fact, it is one of the strongest predictors of community risk. Despite its apparent universality, surprisingly little is understood about how increased income inequality translates into riskier behaviors.

Barnett and Whiteside postulate that income inequality decreases “social cohesion,” the fabric of society that stabilizes sexual relationships [17]. Depending on the society, this likely has different meanings. In the U.S., marriage is a major relationship-stabilizing force. Income inequality decreases marriage rates, perhaps because individuals are more likely to “hold out” for an idealized wealthier partner [65], [66]. Decreased marriage rates, in turn, increase household income inequality, since a greater proportion of individual households are more likely to be funded by a single adult [66]. Thus, income inequality and declining marriage act in a positive feedback loop, entrenching generational poverty [66].

To our knowledge, the clear correlation between declining marriage and rising HIV in the U.S. has not been previously reported. However, sociological studies predict it: despite similar values concerning marriage between whites and blacks, black men and women are increasingly less likely to marry—poverty and unemployment reduce the economic incentive for long-term monogamous commitment [53]. It is important to note that simply encouraging marriage is inadequate—in many low-income countries, HIV risk is associated with *higher* marriage rates, largely because it forces young women into economic dependence on their husbands, while economic forces still encourage both to seek extramarital partners [67], [68], [69]. This suggests that the protective benefits of marriage come from underlying economic securities, not isolated idealization of the institution.

As income inequality grows, the benefits of delaying marriage (“holding out”) rise for all economic strata, but, uniquely amongst the poor, income inequality creates benefits for *earlier* childbearing [69]. Poor women increasingly see no hope in reaching wealthier strata themselves, but may seek emotional fulfillment and potentially economic gain through motherhood instead, entrenching themselves and their children in poverty [69].

Further, greater income inequality encourages the poor to try to emulate wealthier members' growing consumption. In sub-Saharan Africa, women are more likely to engage in concurrent partnerships when they perceive their boyfriend(s) will support them—not necessarily in meeting their basic needs, but in obtaining items such as cell phones [53], [58], [70], [71], [72]. This transactional sex (contrasted with survival sex) can also be observed in poor urban women in the U.S., where receiving financial support from a male sex partner is a leading predictor of partner concurrency [21], [73]. We observed the effects of this in the general HIV association with female-headed households in poverty, and in the uniquely higher HIV incidence amongst blacks in communities where many grandparents are caring for grandchildren.

Income inequality has another consequence important for HIV risk: crime. Most types of crime, particularly violent crime, rise with income inequality, as both the incentive and opportunity for illegal material gain increase (Figure 6) [74]. The resulting loss of males to early death or the corrections system feeds an imbalance in the male:female ratio. Previous work with U.S. county-level data has shown that incarceration-related male:female imbalances do significantly increase the odds of concurrency [22]. Prisons may also act as seeding sites for initial HIV infection: in the U.S., HIV prevalence in prison is roughly four times higher than in the general population [75]. We were unable to collect city-level data regarding incarceration rates; however, crime indices were significantly correlated with HIV amongst black individuals and with heterosexual transmission generally.

### Full circle: male-female imbalances

The disproportionate incarceration of black individuals is not only a function of income and crime disparities, but also of racially biased policy (e.g. “War on Drugs”), and discrimination in both trial and sentencing systems [19], [76]. The resulting systematic disruption of black communities has been called “forced migration” and compared to the Apartheid-era oscillatory migrant labor systems of southern Africa [23]. Correspondingly, we found that male:female imbalances were a uniquely strong predictor of HIV incidence amongst black individuals. Thus, we propose that social cohesion is a victim of both income and social inequalities (Figure 6).

### Limitations

Correlation is not causation: we derive causative principles from numerous, more narrowly focused sociological studies. We cannot be certain that the individuals testing positive for HIV are in the larger group that experiences the correlated distal social determinants. Further, CDC case reports almost certainly underestimate actual HIV cases, since many living with HIV are not tested, especially if they lack health care access. Simultaneously, it may underestimate the incidence denominator, since the Census often undercounts the total number of individuals, particularly amongst the poor and non-white. The likely downward bias of both numerator and denominator increases the chance of accuracy in our HIV incidence estimate.

To ensure comparability, we used only large cities—conclusions may not be generalizable to other areas. Further, measures of residential segregation are notoriously distorted for communities where the minority population is very small: their association factors with HIV incidence amongst black individuals are likely overestimated relative to other factors. However, the trend (Figure 1) is significant.

Finally, while our data do suggest that MSM HIV incidence is lower where SSM is legal, this does not mean that SSM protects against HIV—rather it suggests that the attitudes underlying social acceptance of SSM are protective.

### Conclusions

A rich body of sociological research now exists to explain why some people are more likely resist the HIV prevention message of behavior change. Over the past two decades, researchers have argued over which determining factors—e.g. poverty or wealth, racist/homophobic social structures or cultural practices related to race or sexual orientation—are most important in different times and places so that prevention efforts can be most appropriately targeted. This work is valuable but time and labor-intensive, and is complicated by sample selection. Increasingly, the availability of refined subpopulation-level data permits useful generalizations [12], [29]. We present here a model for generalizing individual risk from community data.

We identified three interrelated, distal social determinants of risk in the U.S.: social inequality, income inequality and lack of economic opportunity. We posit that risk for black individuals in the U.S. is greater because they disproportionately experience all three major distal determinants. Risk amongst MSM is also high, in part due to the physiological, proximal determinant of riskier anal sex, but also exacerbated by social inequality (stigma). If a man engaged in MSM experiences other social determinants, we expect his individual risk would be higher.

This model emerges not just from our study but builds on those presented by other reports [17], [19], [77], and it certainly merits further testing—both sociological and epidemiological. However, it is notable that despite the city-level coarseness of our data, we found many of the same trends predicted by narrower studies, often in other countries. Our findings emphasize the utility of viewing the global HIV epidemic in terms not of race, nor place—but as a set of recurring structural circumstances that select for viral transmission and can be found around the world.
